# Ultrasonographic diagnosis of congenital membranous jejunal stenosis and gastric duplication cyst in a newborn: a case report

**DOI:** 10.1186/s13256-015-0644-2

**Published:** 2015-07-28

**Authors:** Liu Qinghua, Wu Shoucai, Liu Xiaofang, Zhang Xincun, Miao Lili

**Affiliations:** 1 Department of Ultrasound, Jinan Children’s Hospital, Jinan, 250022 China; 2 Department of Ultrasound, Qilu Hospital, Shandong University, Jinan, 250012 China

**Keywords:** Congenital membranous jejunal stenosis, Gastric duplication cyst, Newborn, Ultrasonography

## Abstract

**Introduction:**

Congenital jejunal stenosis and gastric duplication cysts are very rare congenital anomalies of the gastrointestinal tract in the newborn. We present a case of congenital membranous jejunal stenosis associated with gastric duplication cysts, which was diagnosed by ultrasonography. To the best of our knowledge, this is the first report of ultrasonographic diagnosis of congenital membranous jejunal stenosis associated with a gastric duplication cyst in a newborn.

**Case presentation:**

A 1-month-old Chinese baby girl presented with projectile vomiting and hyperpyrexia for 3 days. An upper gastrointestinal contrast study showed incomplete duodenal obstruction; however, ultrasonography revealed congenital membranous jejunal stenosis associated with a gastric duplication cyst. After surgical excision of the jejunal membrane and gastric duplication cyst, she recovered well with no bilious vomiting at the 1-year follow-up.

**Conclusion:**

Ultrasonography is a useful tool for the evaluation of membranous jejunal stenosis and the identification of small, asymptomatic gastrointestinal duplication cysts.

## Introduction

Congenital jejunal stenosis and gastric duplication cysts are very rare congenital anomalies of the gastrointestinal (GI) tract in the newborn; the prevalence rate of jejunoileal atresia and stenosis has been reported to be between 1 in 330 and 0.9 in 10,000 live births, and stenosis occurs in 7 % of cases [1]. The incidence of duplication cysts of the alimentary system is approximately 1 in 4500 live births [2], and gastric duplication cysts represent only 4 to 5 % of these cases [3, 4]. We present a case of congenital membranous jejunal stenosis associated with gastric duplication cysts, which was diagnosed by ultrasonography. The patient underwent surgery and recovered well. To the best of our knowledge, this is the first report of ultrasonographic diagnosis of congenital membranous jejunal stenosis associated with a gastric duplication cyst in a newborn.

## Case presentation

**Fig. 1:**
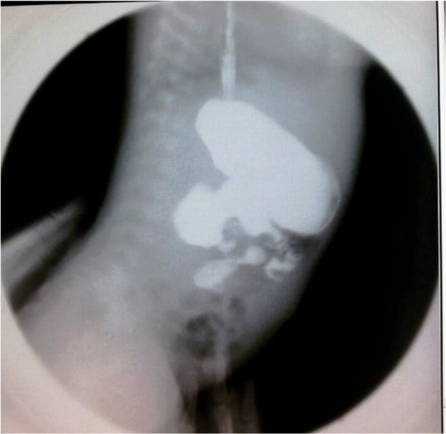
Upper gastrointestinal contrast study showing marked dilatation of the duodenum

**Fig. 2:**
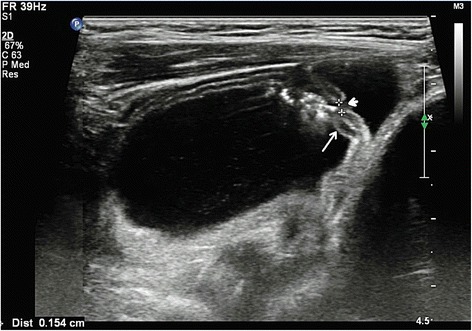
Ultrasonography showing dilatation of the duodenum and proximal jejunum. A membrane (*arrow*) and a pinhole (*small arrowhead*) are seen in the jejunal membrane

**Fig. 3:**
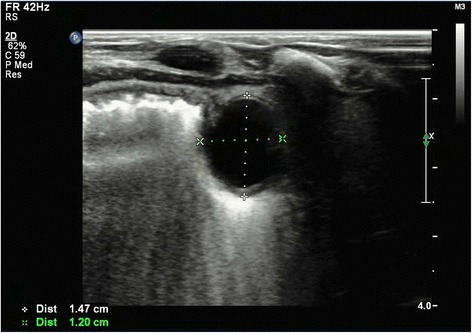
Ultrasonographic image of the gastric duplication cyst. It was 1.5 × 1.4cm and intimately attached at the greater curvature of the stomach

**Fig. 4:**
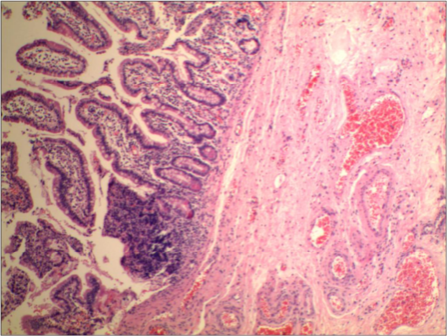
Histopathological examination showing both sides of the resected intestinal membrane covered with intestinal mucosa

**Fig. 5:**
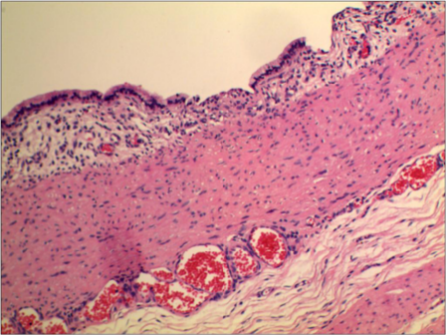
Histological examination of the resected cyst. Gastric mucosa and smooth muscles are seen in the wall of the cyst

A 1-month-old Chinese baby girl presented to our emergency department with vomiting and high-grade fever, which had occurred for 3 days. The fever was not associated with rigors and chills. She presented with a history of intermittent projectile and bilious vomiting after breastfeeding. Vomiting occurred approximately five times each day, at a volume of 10mL at each instance. A general physical examination revealed a temperature of 39 °C, pulse rate of 100/minute, and respiratory rate of 30/minute. On abdominal examination, no abdominal distention, regional tenderness, or rebound was observed, and no masses or viscera could be palpated. Bowel sounds were audible, and other physical examination results were normal. An upper GI contrast study in another hospital showed incomplete duodenal obstruction (Fig. 1). However, to clarify the cause of intestinal obstruction, she subsequently underwent ultrasonographic examination after hospitalization. Ultrasonography showed continuous opening of the pylorus and significant dilatation of her duodenum; her upper jejunum in the left abdomen was approximately 4cm in diameter, and we observed frequent reverse peristalsis. An intraluminal membrane was visible in the dilated distal jejunum site (Fig. 2), and the “billow” in the proximal jejunum could be seen to intermittently move in and out of a pinhole in the mucosal diaphragm. The distal intestine was deflated, and there was an absence of gas; by ultrasonography, a diagnosis of congenital membranous jejunal stenosis was considered. Furthermore, an ultrasound showed a cystic structure sized 2.0 × 1.5cm in the greater curvature in the left hypochondrium (Fig. 3). It was firmly attached at the gastric wall, and the cyst walls consisted of an inner echogenic rim and an outer echogenic rim area. The central area was hypoechoic. A gastric duplication cyst was considered the most likely diagnosis, and a laparotomy was planned after initial stabilization. During surgery, jejunal stenosis situated approximately 15cm from the ligament of Treitz and a diaphragm with a pinhole in the intestinal cavity was found and resected. In addition, surgical excision of the cyst located on the greater curvature of the stomach was performed. A histopathological examination revealed that both sides of the resected intestinal membrane were covered with intestinal mucosa (Fig. 4) and the structure of the gastric mucosa and smooth muscles in the wall of the cyst, confirming the diagnosis of membranous jejunal stenosis and gastric duplication cyst (Fig. 5). Postoperative recovery was uneventful, and she was discharged on the ninth day following surgery. She recovered well with no bilious vomiting at the 1-year follow-up.

## Discussion

In general, the symptoms of patients with congenital anomalies of the alimentary tract present early after birth with recurrent, progressive vomiting. Bilious vomiting indicates obstruction distal to the ampulla of Vater [5], which requires an immediate and correct diagnosis and surgical correction to avoid serious complications. Normally, within 3 hours of birth, the entire small bowel contains gas, whereas gas is observed in the sigmoid colon 8 to 9 hours after birth [6]. In some patients with congenital anomalies of the GI tract causing obstructions, the proximal bowel with lesions exhibits dilatation and effusion, and the distal intestinal cavity has little or no gas, thereby creating favorable conditions for ultrasonic detection of the lesions. Furthermore, ultrasonography of the GI tract is noninvasive, simple, radiation-free, and fairly accurate for the diagnosis of GI obstruction. Therefore, ultrasonography plays a vital role in the evaluation of patients with congenital anomalies of the GI tract. Ultrasonography has been used as an alternative to barium studies in diagnosing certain congenital anomalies of the GI tract, including annular pancreas, hypertrophic pyloric stenosis, enteric duplication cysts, malrotation, and midgut volvulus [5]. However, the use of ultrasonography for the detection and diagnosis of membranous jejunal stenosis has not been previously reported in the literature. A previous study reported that jejunoileal atresia and stenosis are difficult to evaluate [7]. In the present case, ultrasonography revealed the mucosal diaphragm with a pinhole in the jejunum, the duodenum and proximal jejunum loops significantly dilated with fluid, and the distal small intestine deflated with little gas. Based on this case, ultrasonography may have greater importance in the evaluation of membranous jejunal stenosis, as it enables the distinction from atresia and provides excellent anatomic details for correct diagnosis and treatment planning.

Duplication cysts are rare congenital anomalies that can occur in any part of the GI tract, although the most common site is the ileum [3]. Gastric duplication cysts represent only 4 to 5% of duplication cysts and are located along the greater curvature of the stomach, usually in the antrum [3, 4]. Possible causes of gastric duplication cysts include errors in recanalization of the alimentary tract, McLetchie’s theory of traction diverticulum formation caused by endo-ectodermal adhesions, split notochord, and partial twinning [8, 9]. On clinical examination, gastric duplication cysts can present with vague abdominal pain, vomiting, and occasionally a palpable abdominal mass; however, presentation is usually asymptomatic and therefore difficult to diagnose [10]. In the present case, a gastric duplication cyst was incidentally diagnosed by abdominal ultrasonography. Ultrasonography showed a small cystic lesion on the greater curvature of the stomach in the left hypochondrium. Characteristic ultrasonographic features of a GI duplication cyst are a hypoechoic muscular rim surrounding an echogenic mucosa; these features are specific to gastric duplication cysts and are not seen with other cystic lesions [11]. However, in the present case, an upper GI tract contrast study was performed, with no evidence of mass lesions. This case report illustrates the important role of ultrasonography in detecting small, unsuspected duplication cysts.

## Conclusions

Using ultrasonography, we successfully diagnosed congenital membranous jejunal stenosis associated with a gastric duplication cyst in a newborn. This case emphasizes that ultrasonography in a baby with congenital jejunal stenosis was effective as a diagnostic modality. In addition, ultrasonography can be helpful for the accurate identification of small and asymptomatic GI duplication cysts. Ultrasonography is superior to radiography for revealing the cause of some intestinal obstructions, suggesting that babies with bilious vomiting should have a routine ultrasound for diagnosis.

## Consent

Written informed consent was obtained from the patient’s legal guardian for publication of this case report and accompanying images. A copy of the written consent is available for review by the Editor-in-Chief of this journal.

## Abbreviation

GI: Gastrointestinal

## References

[1] Stollman TH, de Blaauw I, Wijnen MH, van der Staak FH, Rieu PN, Draaisma JM, et al. Decreased mortality but increased morbidity in neonates with jejunoileal atresia; a study of 114 cases over a 34-year period. J Pediatr Surg. 2009;44:217–21. 10.1016/j.jpedsurg.2008.10.043 19159746

[2] Potter EL. Pathology of the fetus and newborn. 2nd ed. Chicago: Year Book Medical Publishers; 1961.

[3] Faerber EN, Balsara R, Vinocur CD, de Chadarevian JP. Gastric duplication with hemoptysis: CT findings. AJR Am J Roentgenol. 1993;161:1245–6. 10.2214/ajr.161.6.8249734 8249734

[4] Sieunarine K, Manmohansingh E. Gastric duplication cyst presenting as an acute abdomen in a child. J Pediatr Surg. 1989;24:1152. 10.1016/s0022-3468(89)80101-0 2809990

[5] Gupta AK, Guglani B. Imaging of congenital anomalies of the gastrointestinal tract. Indian J Pediatr. 2005;72:403–14. 10.1007/BF02731737 15973024

[6] Berrocal T, Lamas M, Gutieérrez J, Torres I, Prieto C, del Hoyo ML. Congenital anomalies of the small intestine, colon, and rectum. Radiographics. 1999;19:1219–36. 10.1148/radiographics.19.5.g99se041219 10489177

[7] Sunada K, Yamamoto H, Kita H, Yano T, Sato H, Hayashi Y, et al. Clinical outcomes of enteroscopy using the double-balloon method for strictures of the small intestine. World J Gastroenterol. 2005;11:1087–9. 10.3748/wjg.v11.i7.1087 PMC4250779 15742422

[8] Bonacci JL, Schlatter MG. Gastric duplication cyst: a unique presentation. J Pediatr Surg. 2008;43:1203–5. 10.1016/j.jpedsurg.2008.01.024 18558208

[9] Shinde T, Lindner J, Silverman J, Agrawal R, Dhawan M. Gastric-duplication cyst with an aberrant pancreatic-ductal system: an unusual cause of recurrent abdominal pain. Gastrointest Endosc. 2009;69:377–9. 10.1016/j.gie.2008.04.059 18684455

[10] Prinsloo H, Loveland J, Grieve A, Andronikou S, Valli OM. Gastric duplication cysts as a rare cause of haematemesis: diagnostic challenges in two children. Pediatr Surg Int. 2011;27:1127–30. 10.1007/s00383-011-2864-x 21359665

[11] Barr LL, Hayden Jr CK, Stransberry SD, Swischuk LE. Enteric duplication cysts in children: are their ultrasonographic wall characteristics diagnostic? Pediatr Radiol. 1990;20:326–8. 10.1007/BF02013165 2190153

